# Contribution of Dislocations in SiC Seed Crystals on the Melt-Back Process in SiC Solution Growth

**DOI:** 10.3390/ma15051796

**Published:** 2022-02-27

**Authors:** Sakiko Kawanishi, Hiroyuki Shibata, Takeshi Yoshikawa

**Affiliations:** 1 Institute of Multidisciplinary Research for Advanced Materials, Tohoku University, 2-1-1 Katahira, Aoba-ku, Sendai 980-8577, Japan; hiroyuki.shibata.e8@tohoku.ac.jp; 2 Institute of Industrial Science, The University of Tokyo, 4-6-1 Komaba, Meguro-ku, Tokyo 153-8505, Japan; t-yoshi@iis.u-tokyo.ac.jp

**Keywords:** silicon carbide, in situ observation, melt-back, solution growth, dislocations

## Abstract

The melt-back process has a significant effect on the quality of solution-grown SiC crystals. However, the phenomena surrounding the SiC dissolution into the molten alloy during the melt-back process have not been clarified. In this study, the behavior of 4H-SiC dissolution into molten alloy was investigated by using high-temperature in situ observation and subsequent KOH etching, and the effects of different doping conditions and crystal polarity were studied. Local dissolutions with hexagonal pyramid-shape originating from threading screw dislocation (TSD) were observed on the C face of n-type SiC with light nitrogen doping. Our analysis of their behavior revealed that the process was governed by the spiral dissolution. In addition to the dissolution at TSD, local dissolutions at threading-edge dislocations were observed on the Si face of the same crystal. The shape of the local dissolution at the dislocation was significantly affected by the doping conditions and the polarity of the SiC crystal. This local dissolution may occur during the melt-back process, suggesting that it is important to promote the dissolution while maintaining a smooth interface through the selection of the seed crystal and by keeping the degree of interface undersaturation small.

## 1. Introduction

The selection of a seed crystal material and its orientation is important for the growth of high-quality single crystals. In general, the crystal properties and the surface conditions of the seed crystal have as great an influence at the beginning of growth on the quality of the crystal as the intrinsic nature of the seed material. Therefore, the surface-cleaning process of seed crystals before crystal growth is extremely important in industrial processes.

In the case of silicon carbide (SiC), which is a leading semiconducting material used for high-frequency, high-power, and high-temperature applications [1], homo-epitaxial growth is performed by using 4H-SiC seed substrates. Chemical mechanical polishing (CMP) is generally subjected to give the SiC seed crystal a small surface roughness and reduce bowing. However, a surface treatment before crystal growth is indispensable to remove the subsurface damage remaining even after the CMP. When a 4H-SiC epi-layer is grown by the CVD method, the damaged layer is usually removed by hydrogen etching [2]. In the growth of bulk 4H-SiC single crystals by the solution growth method [3,4,5,6,7,8,9,10,11], which is a potential technique for growing high-quality crystals, the damaged layer is removed by dissolving it through a melt-back process. In addition, without the melt-back process, adhesions of the alloy derived from vapors are present on the surface of the 4H-SiC seed crystal before seed touching the growth solution, which contributes to the formation of various defects, including the solvent inclusions [12]. Therefore, the melt-back process is indispensable for the growth of high-quality 4H-SiC crystals.

Although the effect of the melt-back process has been speculated from SiC crystals after the subsequent growth process [12], the dissolution kinetics of 4H-SiC into molten alloys is not clear. Recently, the authors found that the dissolution process of 4H-SiC causes the formation of dissolution pits originating from dislocations on the Si face [13]. This may form a rough interface before growth and could adversely affect the subsequent growth process. Therefore, in this study, the dissolution behavior of 4H-SiC in a molten alloy was studied in detail to clarify the effect of dislocations in the seed crystals on the melt-back process. The dissolution process of 4H-SiC was investigated by in situ observation method developed by the authors [13,14,15,16] and was correlated to the dislocations utilizing the ex situ anisotropic etching [17,18], which has been used for various crystals [19,20,21]. This allows investigation of the influence of the crystal surface polarities and the doping condition of the 4H-SiC substrate. The preferable condition for the melt-back to maintain the smooth surface was then considered.

## 2. Materials and Methods

**Figure 1:**
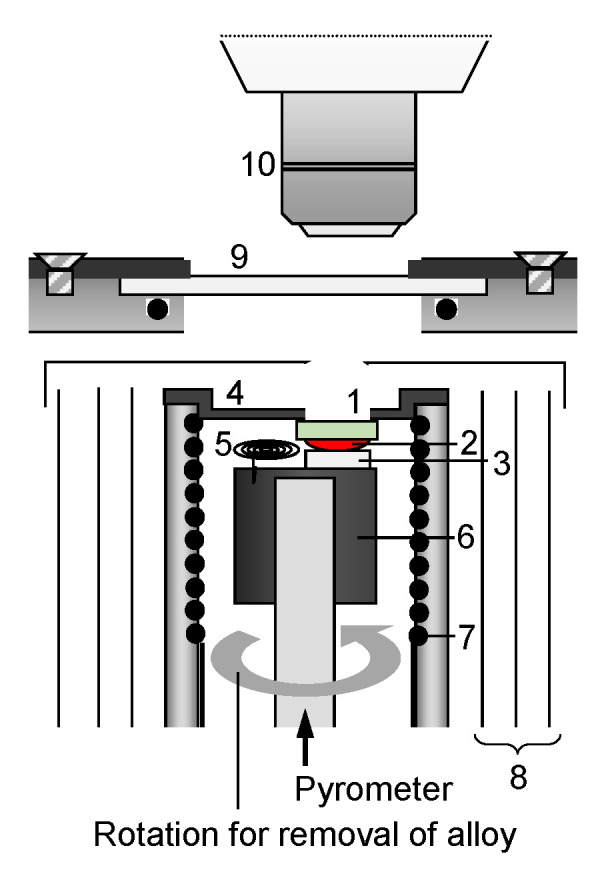
Experimental apparatus for in situ observation of SiC/alloy interface: (1) on-axis 4H-SiC {0001} (80 μm in thickness), (2) molten alloy, (3) Al_2_O_3_ plate, (4) substrate holder, (5) Ta assembly (for removal of alloy), (6) graphite holder, (7) spiral wire heater, (8) reflectors, (9) quartz window, and (10) objective lens.

In situ observation of the dissolution behavior of 4H-SiC substrates into molten Fe-Si alloy was performed to demonstrate the behaviors occurring in the melt-back process. A range of substrates were used with different doping conditions: semi-insulating 4H-SiC with vanadium doping (resistivity: >10^5^ Ω·cm), n-type 4H-SiC with light nitrogen doping (resistivity: 0.207 Ω·cm), and n-type 4H-SiC with heavy nitrogen doping (resistivity: <0.1 Ω·cm). The dissolution behavior of both C and Si faces for each crystal was investigated. The sample configuration of the chamber is shown in Figure 1. A pre-melted Fe-36 mol% Si alloy (30 mg) was placed on an Al_2_O_3_ plate, and an on-axis 4H-SiC {0001} substrate was held above the alloy. Here, the substrate was thinned enough to improve visibility for in situ observation. After evacuating the chamber to below 10^−3^ Pa, He gas was introduced, and the assembly was heated to 1500 K, using a spiral wire heater to melt the alloy. After confirming the formation of a fresh surface of the alloy without any oxide film, the molten alloy was touched to the 4H-SiC substrate by moving it upward. Then the dissolution of SiC started, and the alloy spread on the substrate, owing to the good wettability. After 70 s of SiC dissolution, the alloy was removed from the substrate to conserve the dissolution interface. Here, the removal was achieved by utilizing the capillary phenomena [22,23] by rotating the graphite holder to touch the Ta assembly to the alloy. The entire high-temperature process was observed and recorded by using an in situ observation system that enables simultaneous bright-field and interference observations [14]. After cooling the sample, the as-dissolved SiC substrate was subjected to molten KOH etching [24] to determine the distribution of dislocations from the etch pits.

## 3. Results and Discussion

### 3.1. Dissolution Kinetics of C Face of n-Type 4H-SiC

**Figure 2:**
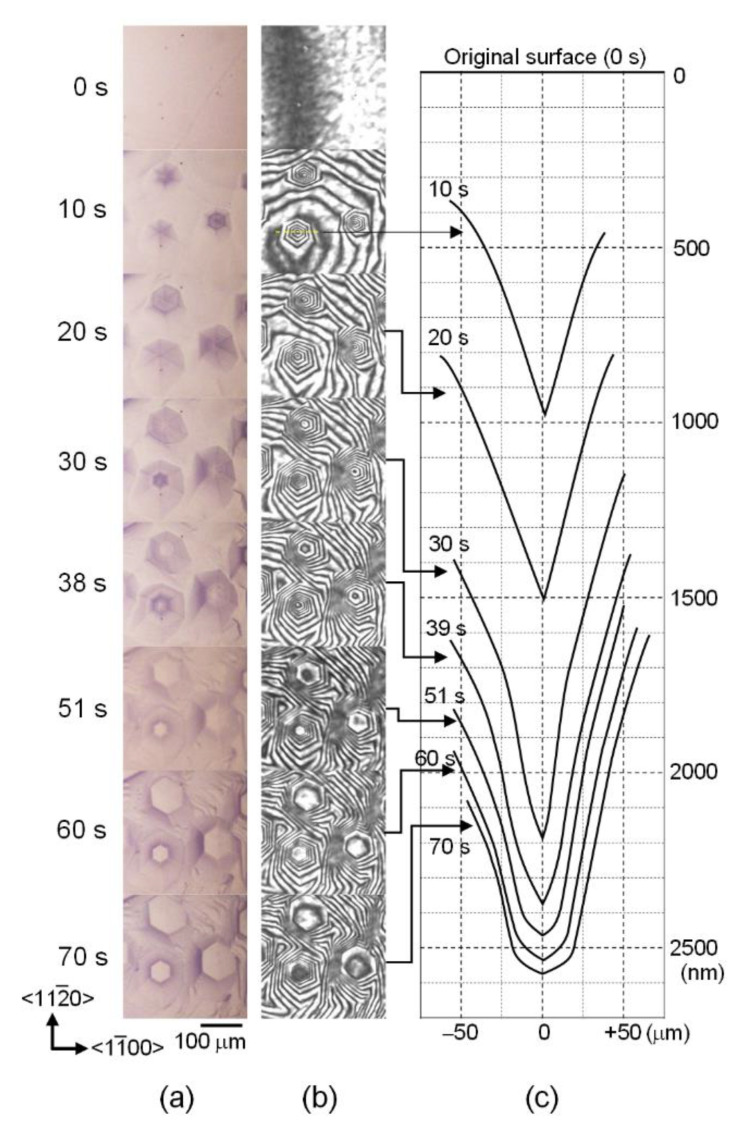
Time evolution of dissolution pit on C face of 4H-SiC: (**a**) bright-field images of 300 × 300 μm^2^, (**b**) interference images for the same area in (**a**), and (**c**) calculated vertical trace of the interface along the <$1\bar{1}00$> direction at a dissolution pit located at the left-bottom part of the images.

**Figure 3:**
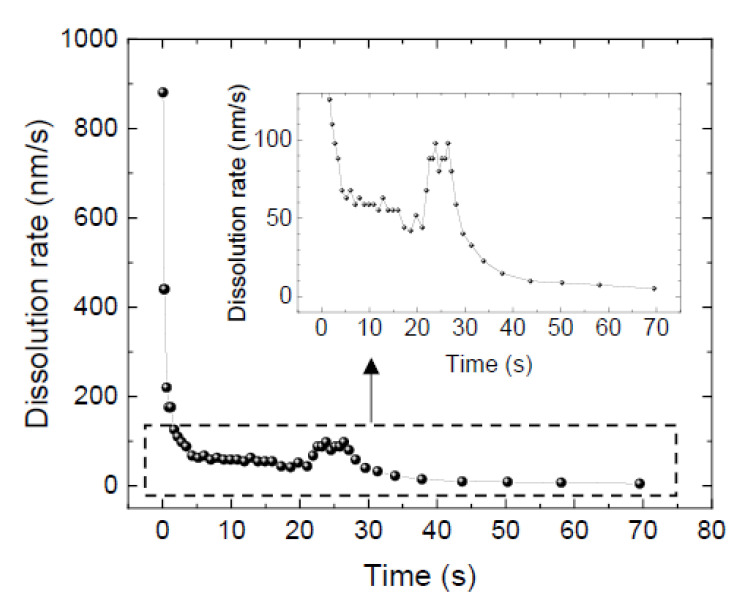
Time dependence of dissolution rate at the center of the pit. The same dissolution pit as in Figure 2 was analyzed.

In the solution growth of bulk 4H-SiC, the C face of n-type 4H-SiC is generally selected as the seed crystal [25]. Therefore, the results obtained by using the C face of n-type 4H-SiC with light nitrogen doping (resistivity: 0.207 Ω·cm) are presented here. After touching the molten alloy to the 4H-SiC substrate, SiC dissolution occurred immediately. Figure 2a,b shows snapshots of the bright-field and interference images during the dissolution. The movie during dissolution is available in Supplementary Video S1. The formation of hexagonal pyramid-shaped dissolution pits is observed. Here, the shade of dissolution pits in the bright-field images reflects the gradient of the pit; the sharper the angle, the darker the image, because of less reflection of the incident light at the interface. The quantitative evaluation of the dissolution height is possible from the interference image [13], and the obtained vertical cross-sectional traces in the <$1\bar{1}00$> direction are shown in Figure 2c. The dissolution rate at the center of the pit (*V*) was analyzed, and the time dependence is shown in Figure 3. Initially, *V* was high (>100 nm/s), and the gradient of the pit was steep. Rapid dissolution occurred because the alloy did not contain carbon at the beginning [13]. At 10 s, the width and depth of the dissolution pit reached >100 μm and >600 nm, respectively (see Figure 2c). The measured *V* gradually decreased as the interface shape became complicated, and the scattering of *V* was also observed. The non-uniform dissolution rate might be attributed to the non-uniform nitrogen distribution and/or the existence of basal plane dislocations. After 50 s, the *V* was less than 10 nm/s as the tip shape became almost flat, and the area expanded.

**Figure 4:**
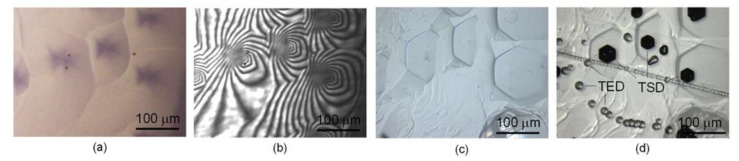
Correlation between dissolution pits on C face of 4H-SiC and dislocations: (**a**) in situ observation image of SiC/alloy interface by bright-field optics, (**b**) corresponding in situ observation image taken by interference optics, (**c**) ex situ image after cooling, and (**d**) ex situ image after KOH etching. Note that the photos for (**c**,**d**) were also taken from the Si face side because the KOH etch pits were formed on the Si face.

Figure 4 shows the correlation between dissolution pits and dislocations. The photos show the in situ high-temperature observation images (Figure 4a,b) and the ex situ images before and after KOH etching (Figure 4c,d) in the same area. The position of the threading screw dislocation (TSD), which KOH etching reveals as a large etch pit, coincides with the position of the hexagonal dissolution pit at a high temperature. Threading-edge dislocations (TED) exist independently of the dissolution pits; therefore, they did not contribute to their formation. Thus, it was presumed that the hexagonal pyramidal dissolution that occurred at high temperatures was a spiral dissolution originating from the TSD in the substrate.

According to the Burton–Cabrera–Frank (BCF) theory for dissolution [26,27,28], the spiral shape is affected by the degree of interface undersaturation (*σ* = (*x*_C_^sat^ − *x*_C_^int^)/*x*_C_^sat^), where *x*_C_^sat^ and *x*_C_^int^ represent the carbon solubility in the alloy and the carbon concentration in the alloy at the SiC/alloy interface, respectively. Although some arguments exist on the BCF theory [29], the step width in the spiral dissolution (*λ*) changes depending on *σ* and satisfies the following relation:

*λ* = 19W*β*/ck*Tσ*, (1)

where W, *β*, c, k, and *T* represent the volume of a Si-C pair (2.076 × 10^−29^ m^3^), step energy in J/m, height of a unit cell (1.0 × 10^−9^ m), the Boltzmann constant (1.38 × 10^−23^ J/K), and temperature in K, respectively. Therefore, *σ* can be obtained from the gradient of the spiral dissolution at high temperatures by assuming a spiral composed of steps with unit cell height. Here, the assumption of *β* = c*γ* was applied to Equation (1) for the evaluation of *σ*, where *γ* is the interfacial energy between SiC and the alloy (0.60 N/m [16]). In addition, the BCF theory allows the following relations:

*V* ∝ *σ*
^2^, *σ* < *σ*_1_ (2)

**Figure 5:**
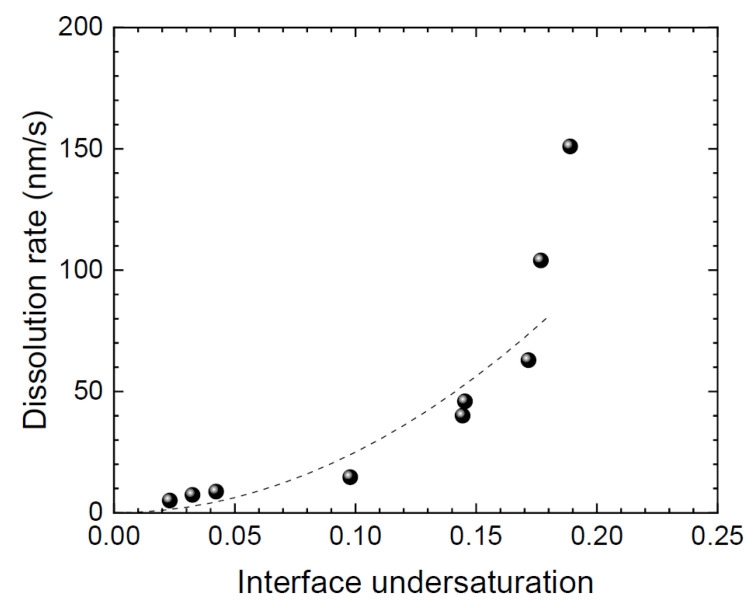
Relation between dissolution rate and interface undersaturation. The dashed curve is a guide to the eye, indicating *y* = *ax*^2^.

*V* ∝ *σ*, *σ* > *σ*_1_, (3)

where *σ*_1_ is a parameter that depends on the length of the diffusion field around the step. Equations (2) and (3) indicate that, when *λ* is wide enough to not interfere with the diffusion field, and *σ* is less than *σ*_1_, *V* is proportion to the square of *σ*. When *σ* is larger, *V* becomes proportional to *σ*, where the solute diffusion is affected by the neighboring steps. Figure 5 shows the relation between *V* and *σ* obtained through in situ observation. The dashed curve illustrates a *V* increases proportional to the square of *σ*. This relationship follows Equation (2), indicating that dissolution is governed by the BCF theory. The deviation at high *σ* was caused by the delay in the shape change attributed to a change in the diffusion field [30]. The implication of the BCF theory is that spiral dissolution would also occur during the melt-back process prior to the solution growth of SiC, and *σ* has a great influence on the interface shape. It is therefore important to conduct the melt-back process under the low *σ* to shift from dissolution to growth while maintaining a smooth interface.

### 3.2. Effect of Doping Condition and Polarity of 4H-SiC

**Figure 6:**
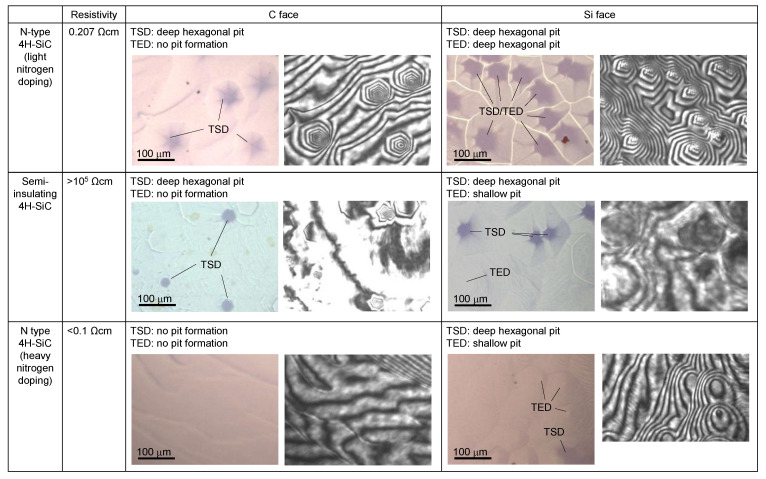
Summaries of correlation between dislocation and dissolution pit together with interface morphologies during SiC dissolution into Fe-Si alloy at ~1500 K. Bright-field image (**left**) and corresponding interference image (**right**) are shown for each condition. All the photos indicate the morphology 10 s after the beginning of dissolution. The relationships between dissolution pit and dislocations were clarified by KOH etching.

Figure 6 summarizes the correlations between the dissolution pits and dislocations in the substrate, together with the corresponding bright-field and interference images during the dissolution at 10 s, when the substrates with different doping conditions and crystal surface polarities were used. On the Si face of the n-type crystal with light nitrogen-doped 4H-SiC (C face discussed in Section 3.1), similar hexagonal pyramid-shape dissolutions were confirmed with a higher number density than observed on the C face. On the Si face, the KOH etching showed that TED also acted as the origin of the dissolution in addition to TSD.

On the C face of the semi-insulating crystal with vanadium doping, TSD was the origin of the spiral dissolution and the formation of hexagonal pits were observed, with no pit formation from TED, as is consistent with the results in Section 3.1. The depth of the spiral dissolution reached >700 nm, and the tip exhibited a steep shape similar to that observed in Section 3.1. Dislocations can be decorated with impurities in crystals with high doping levels [31,32]. Therefore, the steep dissolution shape at the tip might be caused by preferential dissolution near the dislocation core, where the impurity (nitrogen/vanadium) is segregated. No pit formation was confirmed on the C face of the heavily nitrogen-doped crystal. In this case, the isotropic dissolution was dominant compared to the anisotropic dissolution that causes the formation of the dissolution pits. The authors suggest that this is because of the high nitrogen concentration in the whole plane, resulting in no pit formation at the dislocations. From the literature, the shape of the etch pits generated by KOH etching depends on the doping conditions and the surface polarity of the SiC crystals [17,18,24,33]. It was reported that the surface of the Si face of an n-type SiC may turn into p-type conduction through strong band bending and promotes isotropic dissolution [24,34]. The authors suggest that the formation of a Schottky barrier may occur because the electronic state depends on the temperature, properties of the seed crystal, and melt composition, irrespective of the ohmic contact behavior that has been reported for the SiC/Si melt interface at 2143 K [35]. The Schottky barrier results in the formation of a depletion layer that enhances the isotropic dissolution. Therefore, such a difference in the effect of electrochemical etching may also contribute to the difference in the dissolved form.

On the Si face, hexagonal pyramid-shaped dissolutions with steep tips were observed at TSDs for semi-insulating and heavily nitrogen-doped crystals, similar to those observed on the C face. The steep shape could be attributed to the impurities. In these crystals, shallow circular pits (<500 nm in depth) formed by the dissolution at TEDs, exhibiting almost constant gradient. Therefore, no impurity segregation effect was implied at the TED in these crystals.

Regardless of the polarity and doping conditions, all the generated dissolution pits became smoother as the dissolution progressed. The authors suggest that this phenomenon is associated with a decrease in *σ*, as explained in Section 3.1. However, TSD and/or TED can be the origin of dissolution pit formation at high temperatures and can lead to the formation of a rough interface. Thus, to promote the melt-back while maintaining a smooth interface, it is extremely important to maintain a low *σ* for both the Si and C faces.

## 4. Conclusions

The dissolution behavior of 4H-SiC {0001} into molten alloy was studied through in situ studies at ~1500 K, and the contribution of dislocations was clarified through the subsequent KOH etching. The main conclusions are summarized as follows:

(1) When n-type SiC with light nitrogen doping was dissolved, hexagonal spiral dissolutions originating from TSD occurred on the C face, and its behavior followed the BCF theory. The dissolution rate decreased as the degree of interface undersaturation decreased, which is accompanied by approaching a smooth interface. On the Si face of the same crystal, the local dissolutions also occurred at TED.
(2) The dissolution behaviors were also clarified for semi-insulating and heavily nitrogen-doped crystals. The occurrence of local dissolutions at the dislocations and the dissolution shape depended significantly on the doping conditions. Since such local dissolution may occur during the melt-back process, it is important to promote the dissolution while maintaining a smooth interface by selecting the seed crystal and controlling the degree of interface undersaturation to the sufficiently low value.

## Data Availability

The data presented in this study are available upon request from the corresponding author.

## Supplementary Materials

The following supporting information can be downloaded at https://www.mdpi.com/article/10.3390/ma15051796/s1. Video S1: Interface morphology during SiC dissolution into molten alloy at ~1500 K obtained by in situ observation. The snapshots of the trimmed region are shown in Figure 2.
